# Fibromyalgia: Evidence for Deficits in Positive Psychology Resources. A Case-Control Study from the Al-Ándalus Project

**DOI:** 10.3390/ijerph182212021

**Published:** 2021-11-16

**Authors:** Manuel Javier Arrayás-Grajera, Inmaculada Tornero-Quiñones, Blanca Gavilán-Carrera, Octavio Luque-Reca, Cecilia Peñacoba-Puente, Ángela Sierra-Robles, Ana Carbonell-Baeza, Fernando Estévez-López

**Affiliations:** 1 Department of Human Motor Skills and Sports Performance, Faculty of Education, University of Seville, 41013 Seville, Spain; marrayas@us.es; 2 Physical Activity, Promotion of Values and Education, HUM-954 Research Group, University of Huelva, 21007 Huelva, Spain; 3 Department of Integrated Didactics, Faculty of Education, Psychology and Sports Science, University of Huelva, 21007 Huelva, Spain; sierras@uhu.es; 4 Education, Motor Skills and Investigation Huelva Research (EMOTION), HUM643 Research Group, University of Huelva, 21007 Huelva, Spain; 5 PA-HELP “Physical Activity for Health Promotion, CTS-1018” Research Group, Department of Physical Education, Faculty of Education Sciences, University of Cádiz, Puerto Real, 11519 Cádiz, Spain; blanca.gavilan@uca.es; 6 Department of Psychology, Faculty of Health Science, Rey Juan Carlos University, Alcorcon, 28922 Madrid, Spain; octavio.luque@urjc.es (O.L.-R.); cecilia.penacoba@urjc.es (C.P.-P.); 7 MOVE-IT Research Group and Department of Physical Education, Faculty of Education Sciences, University of Cádiz, Puerto Real, 11519 Cádiz, Spain; ana.carbonell@uca.es; 8 Biomedical Research and Innovation Institute of Cádiz (INiBICA) Research Unit, Puerta del Mar University Hospital, 11009 Cádiz, Spain; 9 Department of Child and Adolescent Psychiatry/Psychology, Erasmus MC University Medical Center, 3015 GD Rotterdam, The Netherlands; fer@estevez-lopez.com

**Keywords:** chronic pain, emotional intelligence, fibromyalgia, persistent physical symptoms, resilience (psychological), subjective well-being, vulnerability (psychological)

## Abstract

Positive psychology is the study of positive subjective experience and individual traits. Identifying deficits in positive psychology regarding fibromyalgia may inform targets for management. Therefore, the aim of the present case–control study was to compare the levels of positive affect, negative affect, satisfaction with life, optimism and emotional repair in a large sample of women with fibromyalgia (cases) and age-matched peers without fibromyalgia (controls). This case–control study included 437 women with fibromyalgia (51.6 ± 7.1 years old) and 206 age-matched women without fibromyalgia (50.6 ± 7.2 years old). Participants self-reported their levels of (i) subjective well-being on the Positive and Negative Affect Schedule and the Satisfaction with Life Scale, (ii) dispositional optimism on the Life Orientation Test-Revised and (iii) emotional repair on the Trait Meta-Mood Scale. Women with fibromyalgia showed lower levels of positive affect, satisfaction with life, optimism and emotional repair and higher levels of negative affect. Large effect sizes were found for positive affect, negative affect and satisfaction with life (all, Cohen’s d ≥ 0.80) and small-to-moderate for emotional repair and optimism (both, Cohen’s d ≥ 0.50). Women with fibromyalgia experience deficits of positive psychology resources. Thus, developing tailored therapies for fibromyalgia focusing on reducing deficits in positive psychology resources may be of clinical interest, though this remains to be corroborated in future research.

## 1. Introduction

Fibromyalgia is a chronic disease characterised by widespread musculoskeletal pain and additional physical and psychological symptoms that usually include fatigue, poor sleep quality, stiffness, depression, anxiety and cognitive difficulties [1,2]. As a consequence, people with fibromyalgia report substantial functional limitations, which may lead to high health resource use and lost productivity, resulting in significantly higher costs [3,4]. In fibromyalgia, the exclusive focus on negative aspects has traditionally dominated [5]. However, positive psychology has been defined as the scientific study of positive experiences and positive individual traits [6], which refers to people’s ability to maintain positive functioning in the face of adversity and challenges [7,8].

In fibromyalgia, previous case–control research has extensively focused on the affective sources, such as positive and negative affect [9,10,11], which are the emotional components of subjective well-being, i.e., people’s evaluations of their lives [12]. In comparison to peers without fibromyalgia, those with the disease have reduced levels of positive affect and increased levels of negative affect [9,10,11]. However, scarce attention has been paid to the cognitive-, emotional- and personality-related aspects of positive psychology despite the fact that they may play a key role in adaptation to chronic pain [13]. Satisfaction with life is the cognitive component of subjective well-being, i.e., the cognitive evaluation of one’s life [12,14]. It has been observed that satisfaction with life is low in people with chronic pain [15,16]. Dispositional optimism (herein after referred to as optimism) is the generalised expectancy that good things will happen in the future and bad things will be minimal [17]. In the general population, higher optimism is related to better health [18,19,20]. In fibromyalgia, higher optimism is associated with less perceived barriers to goal attainment during disease flares [21,22]. Emotional repair is the ability that people have to regulate their emotional states themselves [23], enhancing the positive states and minimising those which are negative. In people with chronic pain, higher emotional repair is associated with lower pain [24]. Collectively, it seems that satisfaction with life, optimism and emotional repair may play a role in adaptation to fibromyalgia. However, previous case–control studies have not comprehensively characterised the levels of affective, cognitive and personality aspects of positive psychology in fibromyalgia. Addressing this gap in the knowledge may help to develop tailored therapies focused on reducing fibromyalgia deficits in positive psychology and, consequently, the severity of the disease.

Therefore, the aim of the present case–control study was to compare the levels of positive affect, negative affect, satisfaction with life, optimism and emotional repair in a large sample of women with fibromyalgia (cases) and age-matched women without fibromyalgia (controls). On the basis of the previous evidence, the hypothesis of the present study was that, in comparison to controls, women with the disease would have more unfavourable scores in all of these positive psychology resources.

## 2. Materials and Methods

### 2.1. Participants

Participants from the eight provinces of Andalusia (southern Spain) were recruited for the present case–control study through local fibromyalgia associations via e-mail, letter, telephone or university press. Additionally, participants with fibromyalgia (cases) were asked to recruit non-fibromyalgia (control) participants with a similar age and socio-demographic characteristics to conduct comparisons between groups. All potential participants (*n* = 960) gave their written informed consent after receiving detailed information about the study aims and procedures before taking part in the study. The inclusion criteria for participants with fibromyalgia were: (1) to be an adult woman (aged 18 to 65 years old); (2) to have a medical diagnosis of fibromyalgia from a rheumatologist (participants were requested to provide their medical records to confirm their diagnosis); and (3) to meet the 1990 American College of Rheumatology (ACR) fibromyalgia criteria [25,26]. The inclusion criteria for control participants were: (1) to be an adult woman (aged 18 to 65 years old); and (2) not to meet the 1990 ACR fibromyalgia criteria [25]. Additionally, fibromyalgia or control participants with either acute/terminal illness (such as cancer, stroke, recent cardiopathy, severe coronary disease, schizophrenia or any other disabling injury) or severe cognitive impairment, as defined by a score of less than ten on the Mini-Mental State Examination (MMSE) [27,28], were excluded. All tests were performed by the same trained group of researchers to reduce inter-examiner error. The study was reviewed and approved by the Ethics Committee of the “Hospital Virgen de las Nieves” (Granada, Spain), registration number: 15/11/2013-N72. The ethical guidelines of the Declaration of Helsinki (modified in 2000) were followed.

### 2.2. Measures

The MMSE [27,28] was used as a screening test to determine whether a participant experienced severe cognitive impairment (i.e., an exclusion criterion). The MMSE assesses five areas of cognitive function: orientation, immediate memory, attention/concentration, delayed recall and language.

Socio-demographic data were recorded using a self-report questionnaire in which participants indicated their date of birth, marital status (i.e., married, single, separated, divorced and widowed), education level (i.e., unfinished studies, primary studies, secondary studies and university studies), time since fibromyalgia diagnosis (<1 year, 1–5 years and >5 years) and time since first symptoms until fibromyalgia diagnosis (<1 year, 1–5 years and >5 years).

A physical examination of tender points according to the 1990 ACR fibromyalgia criteria [25,26] was performed using a standard pressure algometer (FPK 20; Wagner Instruments, Greenwich, CT, USA). This examination was performed by fully trained researchers. Those participants who (i) reported widespread musculoskeletal pain for at least 3 months and (ii) reported pain at a pressure of ≤4 kg/cm^2^ in at least 11 tender points were classified as meeting the 1990 ACR fibromyalgia criteria.

The Positive and Negative Affect Schedule (PANAS) [29,30,31] is a 20-item questionnaire with a 5-point Likert scale (from 1 = “very slightly or not at all” to 5 = “extremely”) that assesses two subscales, positive and negative affect. The scores of the PANAS range from 10 to 50, where higher scores reflect greater positive affect or negative affect. We used the trait time-frame version of the PANAS, i.e., “in general” directions. The 2-factor structure of the PANAS has shown satisfactory psychometric properties in fibromyalgia [31]. In the present study, Cronbach’s alpha was 0.91 for positive affect and 0.91 for negative affect.

The Satisfaction with Life Scale (SWLS) [32,33] is a 5-item questionnaire with a 7-point Likert scale (from 1 = “strongly disagree” to 7 = “strongly agree”). The score of the SWLS ranges from 5 to 35, where a higher score reflects greater satisfaction with life. In the present study, Cronbach’s alpha for satisfaction with life was 0.86.

The Life Orientation Test-Revised (LOT-R) [17,34,35,36] is a 10-item questionnaire with a 5-point Likert scale (from 0 = “strongly disagree” to 4 = “strongly agree”). LOT-R is composed of three positively worded items (e.g., “I’m always optimistic about my future”), three negatively worded items (e.g., “I hardly ever expect things to go my way”) and four fillers that are not considered in the LOT-R score. The score of the LOT-R ranges from 0 to 24, where a higher score reflects greater dispositional optimism. In the present study, Cronbach’s alpha for optimism was 0.70.

The emotional repair scale of the Trait Meta-Mood Scale (TMMS-ER) [37,38] is an 8-item questionnaire with a 5-point Likert scale (from 1 = “strongly disagree” to 5 = “strongly agree”). The score of the emotional repair scale of the TMMS ranges from 8 to 40, where a higher score reflects the perceived ability to enhance positive emotional states and minimise negative ones. In the present study, Cronbach’s alpha for emotional repair was 0.90.

### 2.3. Procedure

Assessments took place over three consecutive days. On day 1, the MMSE was conducted, socio-demographic data were collected and a tender point examination was conducted. On day 2, participants filled out the PANAS, SWLS, LOT-R and TMMS-ER questionnaires at home without supervision. On day 3, participants returned the questionnaires to the research team who reviewed all of them, and potential doubts were resolved.

### 2.4. Statistical Analysis

Descriptive statistics include means and standard deviations for continuous variables as well as frequencies and percentages for categorical variables. Internal consistency of the instruments was assessed by Cronbach’s α coefficient with acceptable values ≥0.70 [39]. Before main analyses, *t*-test and chi-square test analysis (in continuous and categorical variables, respectively) were used to identify differences between groups (i.e., participants with and without fibromyalgia) in the potential socio-demographic confounders; namely age, marital status and education level. Statistical differences in education level between women with and without fibromyalgia were found and, thus, it was included as a covariate in all the analyses.

Multivariate analysis of covariance (MANCOVA) was conducted to compare the mean scores of fibromyalgia and non-fibromyalgia groups on positive psychology resources. In MANCOVA, the group was entered as the independent variable; all the positive psychology resources were entered as dependent variables and education level was entered as a covariate. The effect size was calculated using Cohen’s d (standardised mean differences) to compare all the positive psychology variables. Cohen’s d values were interpreted as small (~0.2), medium (~0.5) or large (~0.8) effects [40].

All analyses were performed using Statistical Package for Social Sciences (IBM SPSS Statistics for Windows, version 26.0; Armonk, NY, USA), and the level of statistical significance was set at α = 0.05 (two-tailed).

## 3. Results

**Figure 1:**
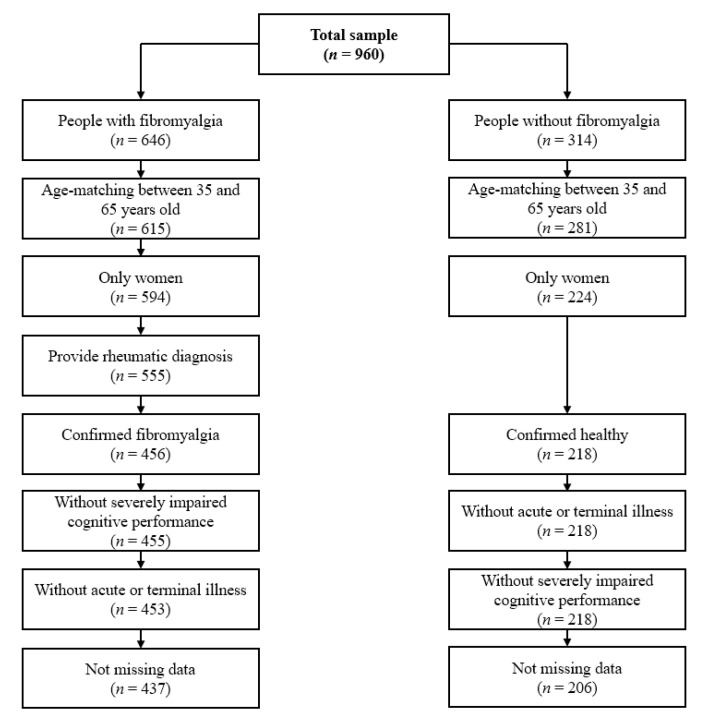
Flowchart of the participants.

Figure 1 shows the flowchart of the participants. Table 1 shows the characteristics of the 643 participants (437 women with fibromyalgia and 206 non-fibromyalgia women).

**Figure 2:**
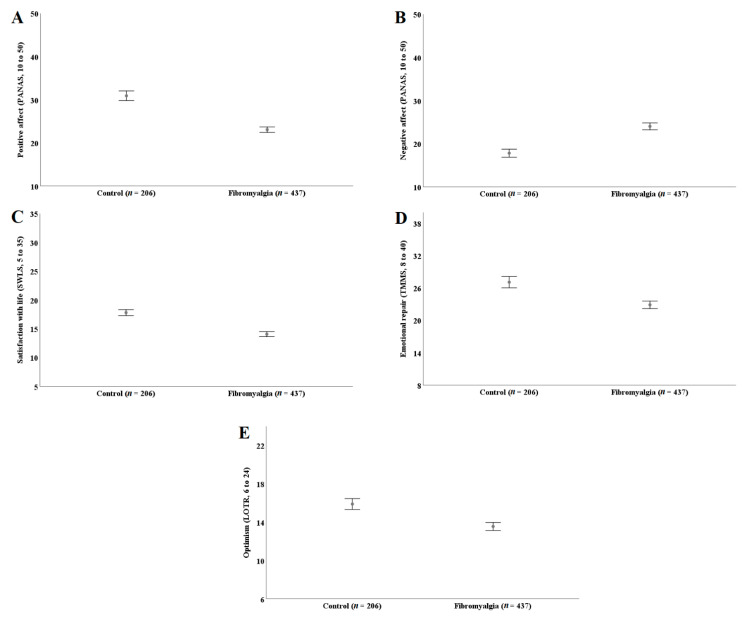
Panel (**A**–**E**): Levels of positive affect, negative affect, satisfaction with life, emotional repair and optimism in women with and without fibromyalgia. PANAS: Positive and Negative Affect Schedule. SWLS: Satisfaction with Life Scale. TMMS: Trait Meta-Mood Scale. LOT-R: Life Orientation Test-Revised.

A significant effect of the group emerged on positive psychology resources (V = 0.241, F (5, 636) = 40.43, *p* < 0.001) adjusting for age and education level. Figure 2 (panel A to E) shows that women with fibromyalgia have lower levels of positive affect (F(1, 640) = 154.47, panel A), satisfaction with life (F(1, 640) = 100.23, panel C), emotional repair (F(1, 640) = 40.33, panel D) and optimism (F(1, 640) = 36.78, panel E), and higher levels of negative affect (F(1, 640) = 82.59, panel B) compared to women without fibromyalgia (all *p*’s < 0.001). The effect sizes were medium (0.56 for emotional repair and 0.53 for optimism) and large (1.07 for positive affect, −0.79 for negative affect and 0.87 for satisfaction with life).

## 4. Discussion

The aim of the present study was to compare the levels of positive affect, negative affect, satisfaction with life, emotional repair and optimism between women with fibromyalgia and age-matched peers without fibromyalgia (cases and controls, respectively). As we hypothesised, women with fibromyalgia experienced deficits in positive psychology resources. Large effect sizes were found for three components of subjective well-being (namely positive affect, negative affect and satisfaction with life; all, Cohen’s d ≥ 0.80). Effect size ranged from small to medium for emotional repair and optimism. The comprehensive characterisation of the positive psychology of women with fibromyalgia provided in the present study may inform the development of tailored therapies for this disease. For instance, therapies targeted at improving positive affect may be a priority in fibromyalgia in order to improve the markedly high deficits observed in these resources of positive psychology.

In the present study, women with fibromyalgia experienced lower positive affect and higher negative affect than women without fibromyalgia, which concurs with previous findings from smaller samples [10,41,42,43,44,45]. Indeed, Zautra et al. [9] and Hassett et al. [11] underlined that, in women with fibromyalgia, positive affect and negative affect are more deteriorated than in women without fibromyalgia (healthy peers) or women with other rheumatic conditions (e.g., osteoarthritis). Previous case–control studies did not analyse satisfaction with life, which is the cognitive component of subjective well-being. The present findings confirmed that, in comparison to peers without fibromyalgia, women with the disease have lower levels of satisfaction with life. This finding is in line with several previous studies on chronic musculoskeletal pain [15,46,47,48]. Thus, it seems that fibromyalgia, similarly to other chronic pain conditions, imposes a burden on levels of subjective well-being in general and, particularly, on positive affect.

To the best of our knowledge, this is the first case–control study analysing levels of emotional repair and optimism in fibromyalgia, and it demonstrates that women with the disease also have deficits in cognitive- and personality-related aspects of positive psychology. Reduced levels in emotional repair [24] and optimism [49] have been previously observed in other populations with chronic pain. Thus, our findings extend previous observations from other chronic pain populations to fibromyalgia.

Previous studies concluded that more favourable levels of positive and negative affect [9,10,11], satisfaction with life [50], emotional repair [51] and optimism [22] are associated with better adaptation to fibromyalgia (e.g., reduced pain [45], fatigue [52] and disease severity [44]). Therefore, they are considered resources of adaptation to fibromyalgia. As a group, women with fibromyalgia showed the largest deficit in positive affect and large deficits in negative affect and satisfaction with life. Thus, therapies may focus on enhancing subjective well-being as a priority when aiming to increase positive psychology. The results of different studies collected in a previous meta-analysis revealed that positive psychology interventions do indeed significantly enhance well-being [53]. It is also important to note that fibromyalgia is a heterogeneous population [54] and that decisions about the aims of any therapy should be defined by a process of shared decision making with the person who has the disease [55,56].

Some limitations of the present study need to be mentioned. This study was conducted only on women and the findings are not generalisable to men. The study was designed cross-sectionally. Thus, it precludes the possibility of determining the causality of the associations under study. Additionally, a group of people with a chronic disease similar to fibromyalgia (e.g., chronic fatigue syndrome or bowel syndrome) was not included. Thus, it is unknown whether our findings are specific to fibromyalgia or generalisable to other similar diseases. Future longitudinal research including women and men as well as another disease control group is of interest. The strengths of the present study were the inclusion of (i) a large sample, (ii) a comprehensive assessment of the affective-, cognitive- and personality-related aspects of positive psychology and (iii) a corroboration by the research team that cases met the diagnosis of fibromyalgia by a physical examination and controls did not.

## 5. Conclusions

Women with fibromyalgia have lower values of positive affect, satisfaction with life, emotional repair and optimism, and higher values of negative affect than women without fibromyalgia. Large effect sizes were found for positive affect, negative affect and satisfaction with life. Thus, developing tailored therapies for fibromyalgia focusing on reducing deficits in positive psychology resources may be of clinical interest, though this remains to be corroborated in future research.

## Figures and Tables

**Table 1:** Characteristics of the participants (*n* = 643).

Characteristics	Fibromyalgia (*n* = 437)	Control (*n* = 206)	*p*-Value
Age, mean (SD)	51.6 (7.1)	50.6 (7.2)	0.081
Education level, *n* (%)			0.004
Unfinished studies	40 (9.2)	11 (5.3)	
Primary studies	210 (48.1)	81 (39.3)	
Secondary studies	126 (28.8)	65 (31.6)	
Tertiary studies	61 (14.0)	49 (23.8)	
Marital status, *n* (%)			0.700
Married	332 (76.0)	149 (72.3)	
Single	34 (7.8)	21 (10.2)	
Separated/divorced	50 (11.4)	26 (12.6)	
Widow	21 (4.8)	9 (4.4)	
Missing data	0 (0)	1 (0.5)	
Time since fibromyalgia diagnosis, *n* (%)			
<1 year	28 (6.6)		
1–5 years	147 (34.6)		
>5 years	250 (58.8)		
Missing data	12 (2.7)		
Time since first symptoms until fibromyalgia diagnosis, *n* (%)			
<1 year	41 (9.6)		
1–5 years	181 (42.6)		
>5 years	203 (47.8)		
Missing data	12 (2.7)		

## Data Availability

Data are available under reasonable request from M.J.A.-G., I.T.-Q., A.C.-B. and F.E.-L.
